# Dose-related liver injury of Geniposide associated with the alteration in bile acid synthesis and transportation

**DOI:** 10.1038/s41598-017-09131-2

**Published:** 2017-08-21

**Authors:** Jingzhuo Tian, Jingjing Zhu, Yan Yi, Chunying Li, Yushi Zhang, Yong Zhao, Chen Pan, Shixie Xiang, Xiaolong Li, Guiqin Li, John W Newman, Xiaoyi Feng, Jing Liu, Jiayin Han, Lianmei Wang, Yue Gao, Michael R. La Frano, Aihua Liang

**Affiliations:** 1 Institute of Chinese Materia Medica, China Academy of Chinese Medical Sciences, Beijing, China; 2 NIH West Coast Metabolomics Center, Davis, CA95616 USA; 3 United States Department of Agriculture, Agricultural Research Service, Western Human Nutrition Research Center, Davis, CA95616 USA; 4 Department of Nutrition, University of California-Davis, Davis, CA95616 USA; 5 Beijing Institute of Radiation Medicine, Beijing, China; 6 Department of Food Science and Nutrition, California Polytechnic State University, San Luis Obispo, CA USA

## Abstract

*Fructus Gardenia* (FG), containing the major active constituent Geniposide, is widely used in China for medicinal purposes. Currently, clinical reports of FG toxicity have not been published, however, animal studies have shown FG or Geniposide can cause hepatotoxicity in rats. We investigated Geniposide-induced hepatic injury in male Sprague-Dawley rats after 3-day intragastric administration of 100 mg/kg or 300 mg/kg Geniposide. Changes in hepatic histomorphology, serum liver enzyme, serum and hepatic bile acid profiles, and hepatic bile acid synthesis and transportation gene expression were measured. The 300 mg/kg Geniposide caused liver injury evidenced by pathological changes and increases in serum alanine aminotransferase (ALT), aspartate aminotransferase (AST), alkaline phosphatase (ALP) and γ-glutamytransferase (γ-GT). While liver, but not sera, total bile acids (TBAs) were increased 75% by this dose, dominated by increases in taurine-conjugated bile acids (t-CBAs). The 300 mg/kg Geniposide also down-regulated expression of Farnesoid X receptor (FXR), small heterodimer partner (SHP) and bile salt export pump (BSEP). In conclusion, 300 mg/kg Geniposide can induce liver injury with associated changes in bile acid regulating genes, leading to an accumulation of taurine conjugates in the rat liver. Taurocholic acid (TCA), taurochenodeoxycholic acid (TCDCA) as well as tauro-α-muricholic acid (T-α-MCA) are potential markers for Geniposide-induced hepatic damage.

## Introduction

**Figure 1:**
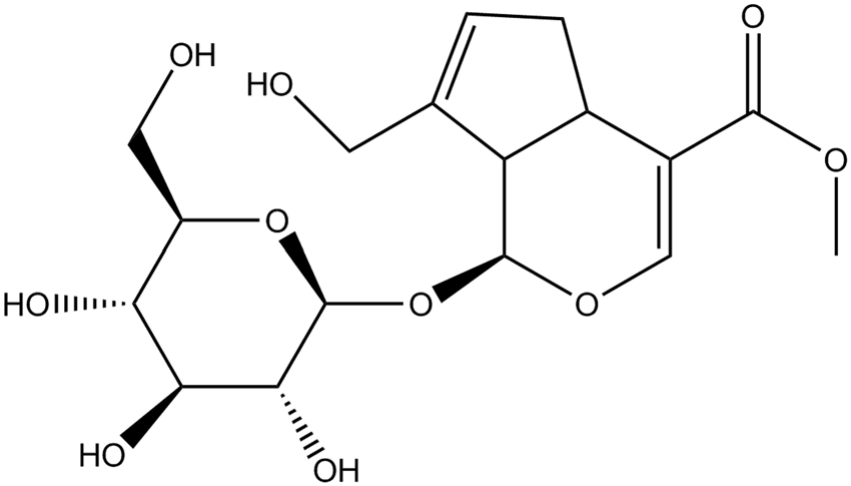
Chemical structure of Geniposide.

Chinese material medica (CMM) used worldwide as a medicine or dietary supplements has greatly increased in recent years. Since most of CMM have not been fully evaluated in toxicological researches, sufficient scientific data is not available to guide their safe clinical use. This situation has led to some irrational clinic applications of CMM resulting in adverse reactions and toxicity^1, 2^. FG, containing the major active constituent Geniposide^3, 4^ (chemical structure of Geniposide shown in Fig. 1), has been widely used in China for the treatment of various diseases including jaundice^4^, diarrheal, and gastroenteritis^5^. No clinical reports regarding FG toxic reactions have been reported in literatures to date. However, since FG is typically used in formulation with other CMM, it is difficult to recognize the adverse effect of this single herb. Moreover, CMM-induced liver injury is difficult to observe during the period of treatment due to a lack of overt symptoms even when liver injury is present. Nevertheless, animal studies have shown that an extract of FG or Geniposide itself can cause hepatotoxicity in rats^6, 7^, and that oxidative stress was likely involved in Geniposide-induced hepatic damage^8^. It must be noted that the doses of Geniposide to cause liver injury were ≥280 mg/kg in rats which is substantially higher than the doses generally used for pharmacodynamics in animal studies (25 mg/kg to 100 mg/kg)^7, 9, 10^. Since Geniposide levels in FG vary from 1.8–6%, Geniposide overexposure with potential hepatotoxic outcomes are possible^11–13^. Therefore, knowledge of Geniposide-induced liver injury and its hepatotoxic mechanism are needed to allow the safe clinical use of the CMM.

Bile acids play essential roles in regulating cholesterol, triglyceride, and glucose homeostasis^14^. The primary bile acids (PBAs), such as chenodeoxycholic acid (CDCA) and cholic acid (CA), are synthesized from cholesterol in hepatocytes. Rodents also synthesize α-muricholic acid (α-MCA) and β-muricholic acid (β-MCA)^15^. Secondary bile acids (SBAs) including lithocholic acid (LCA), ursodeoxycholic acid (UDCA) and deoxycholic acid (DCA) are derived from PBAs by microbial flora in the large intestine^16^. PBAs and SBAs can be transformed into conjugated bile acids (CBAs), including t-CBAs and glycine-conjugated bile acids (g-CBAs). Approximately 95% of the bile acids excreted into the bile duct from hepatocytes are reabsorbed in the terminal ileum and returned back to the liver for further biliary secretion^17^. Some liver diseases and drug-induced liver injuries can disturb the synthesis and clearance of hepatic bile acids potentially resulting in alteration of the composition and concentration of bile acids in liver and sera. The consequential bile acid accumulation in liver can result in hepatotoxicity and even lead to cirrhosis and hepatic necrosis^18, 19^. Hence, bile acids have been considered as biomarkers of hepatic diseases^20, 21^. FXR and various hepatic transporters such as the Na^+^-dependent taurocholic cotransporting polypeptide (NTCP), BSEP, multidrug resistance associated protein 2 & 3 (Mrp2, Mrp3) play pivotal roles in regulating bile acid homeostasis via regulation of synthesis, transportation of bile acids^18, 22–29^ and their proper function of this excretion is critical to prevent bile acid mediated hepatotoxicity.

In the present study, we explored Geniposide induced hepatotoxicity in rats and its effect on bile acid levels and metabolism, to search for potential markers and elucidate the mechanism associated with Geniposide-induced liver injury.

## Results

### Physical effects and liver weights

Manifestations, including diarrhea, weakness and weight loss, and one death rat were observed only at the 300 mg/kg Geniposide dose and relative liver weight (g/100 g body weight) were increased after 3 days (data not shown). There were no abnormal signs in the rats in 100 mg/kg Geniposide group.

### Geniposide caused liver injury at high dose level

**Figure 2:**
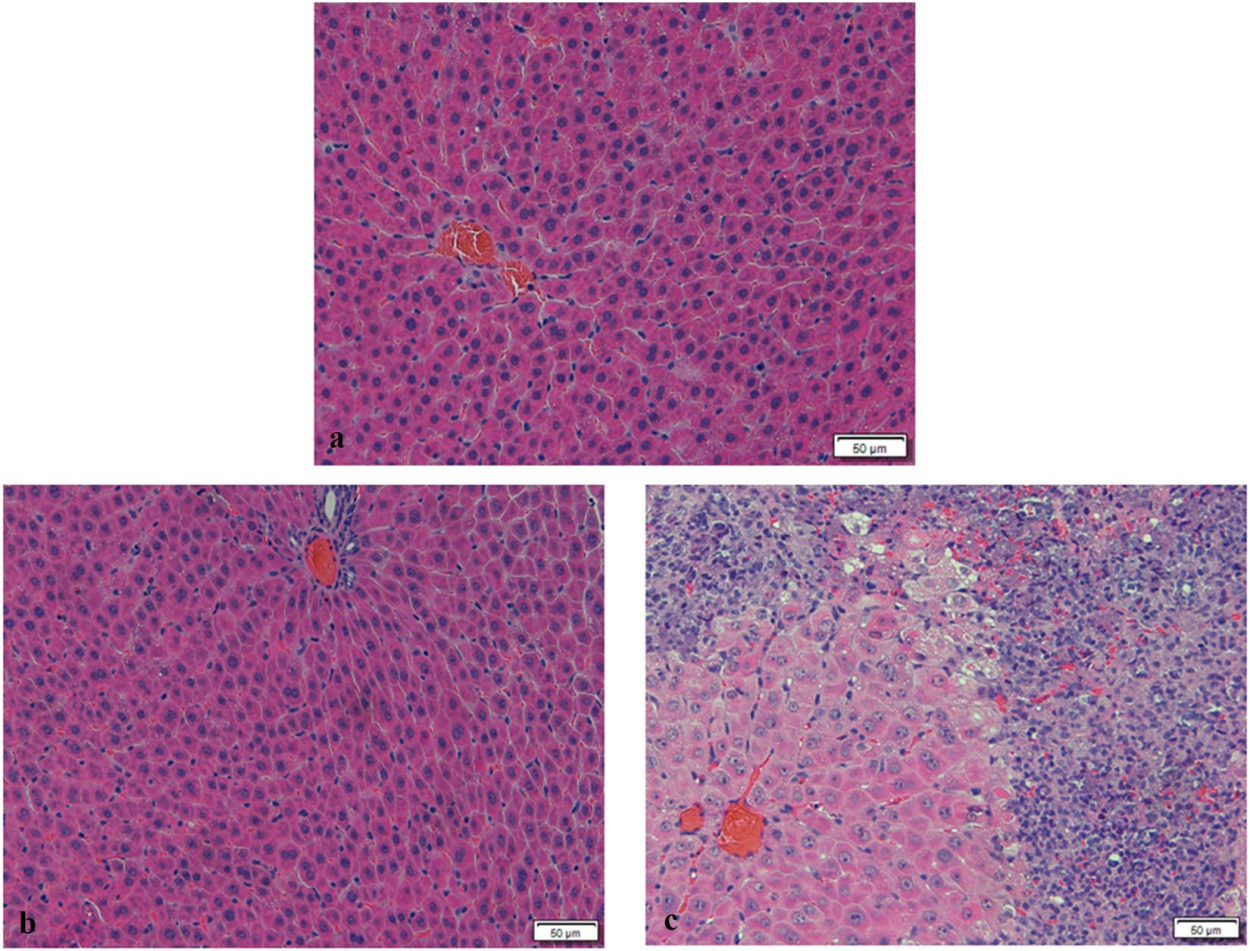
Histomorphological changes in livers of rats with or without Geniposide treatment. Paraffin-embedded liver sections were stained with haematoxylin and eosin (HE). (**a**) Control, (**b**) Geniposide 100 mg/kg, (**c**) Geniposide 300 mg/kg.

After rats 300 mg/kg Geniposide treatment, the serum concentration of ALT, AST, and ALP increased significantly (*p* < *0.05*) (Table 1). In addition, γ-GT and cholesterol (CHO) were increased at both 100 and 300 mg/kg doses (*p* < *0.05*) (Table 1). A decrease of total bilirubin (TBIL) was noted with Geniposide at the 100 mg/kg (*p* < *0.001*) (Table 1), but not 300 mg/kg (*p* = *0.2*) dose. Histological findings included hepatocyte swelling with degeneration or necrosis, fat droplets in hepatocytes, and lymphocytes infiltration in the 300 mg/kg Geniposide group (Fig. 2c). Histological abnormalities were not observed in the 100 mg/kg group (Fig. 2b). Therefore, the high dose of Geniposide caused liver injury in rats.

**Table 1:** Serum biochemical values of ALT, AST, ALP,γ-GT, CHO, TBIL after rats treated with Geniposide for 3 days.

	Control	Geniposide	
	—	100 mg/kg	300 mg/kg
ALT (U/L)	25.00 ± 4.87	26.38 ± 10.68	291.00 ± 294.36*
AST (U/L)	131.50 ± 16.62	108.75 ± 18.39*	474.86 ± 283.96**
ALP (U/L)	278.63 ± 42.08	277.38 ± 40.95	455.71 ± 169.89*
γ-GT (U/L)	1.17 ± 1.68	8.44 ± 7.25*	15.76 ± 15.91*
CHO (mmol/L)	1.75 ± 0.53	2.33 ± 0.50*	2.60 ± 0.37**
TBIL (μmol/L)	0.83 ± 0.25	0.01 ± 0.04***	2.40 ± 5.61

Data are presented as means ± SD of 7–8 rats. *p < *0.05*, **p < *0.01*, ***p < *0.001*, compared with the control group.

### Multivariate statistical analysis of bile acids in sera and livers

**Figure 3:**
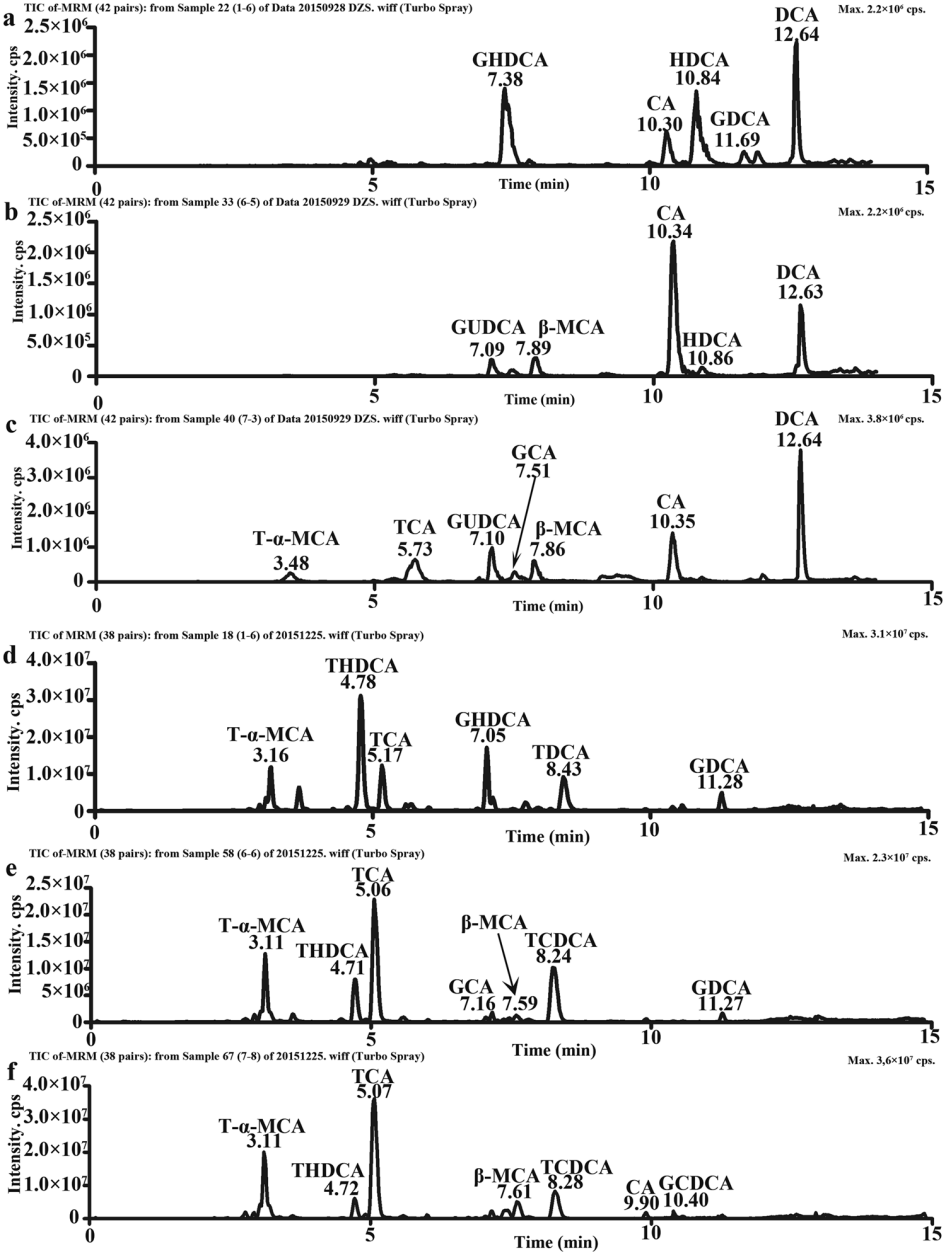
UPLC-MS/MS chromatogram of bile acids in sera and livers. Serum samples in control group (**a**), Geniposide 100 mg/kg group (**b**), Geniposide 300 mg/kg group (**c**) and hepatic samples in control group (**d**), Geniposide 100 mg/kg group (**e**), Geniposide 300 mg/kg group (**f**).

**Figure 4:**
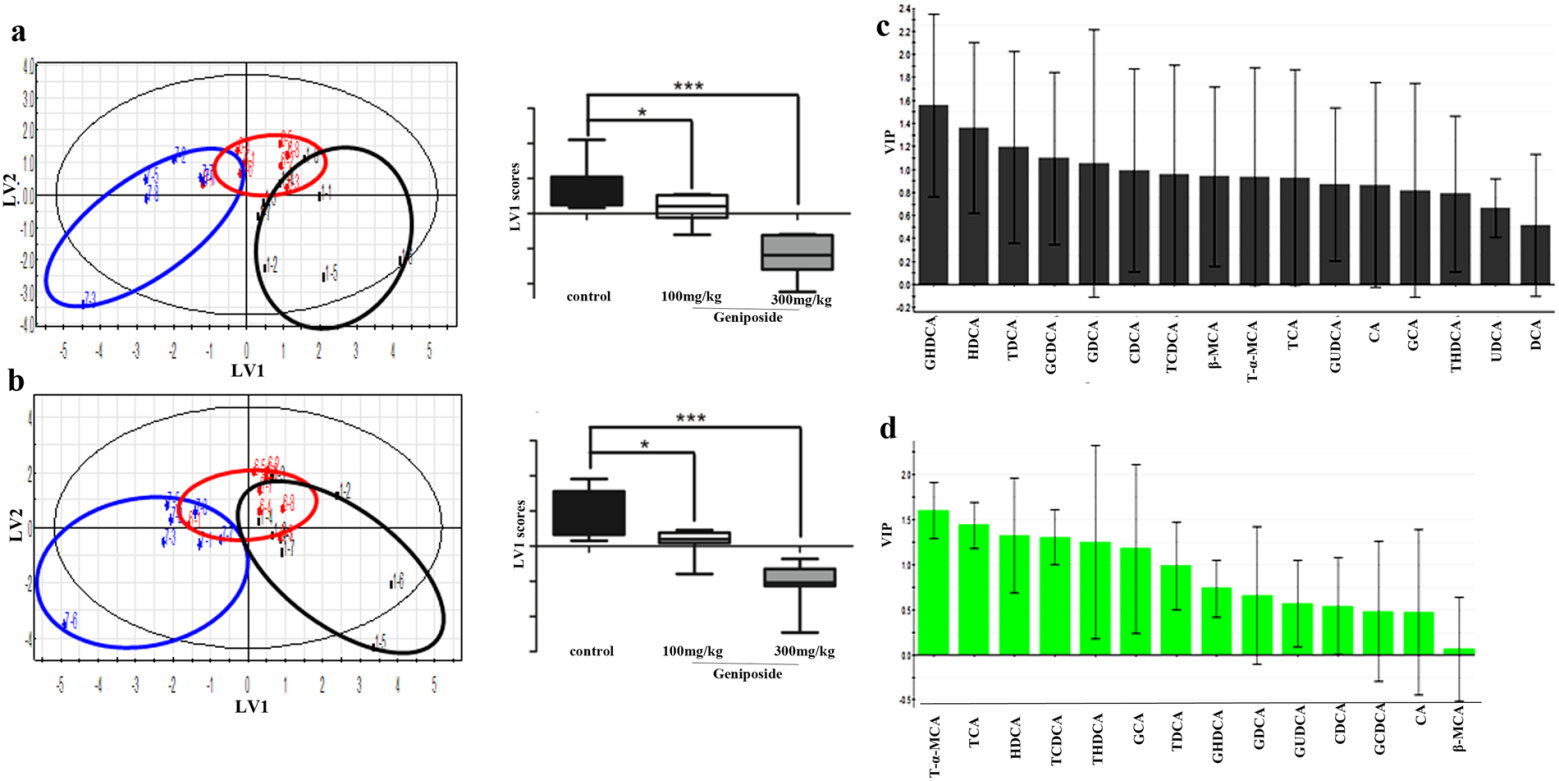
Multivariate data analysis of bile acid profiles in sera and liver. The PLS-DA score plots demonstrated complete separation of samples between groups in sera (**a**) and liver (**b**). The black circles represented the control, while the red and blue circles represented the Geniposide 100 mg/kg and 300 mg/kg group respectively, as indicated on the plots. According to PLS-DA score plots, LV1 scores in sera and liver were presented, respectively. The VIP plots of PLS-DA highlighted the discriminatory species in sera (**c**) and liver (**d**). *p < *0.05*, ***p < *0.001*, compared with the control group.

Representative UPLC-MS/MS chromatograms of bile acids detected in the sera and livers are shown in Supplementary Fig. S1. Sixteen bile acids, including 5 t-CBAs, 5 g-CBAs and 6 unconjugated bile acids (UCBAs) were quantified in control and Geniposide-treatment groups (Fig. 3a–f). An initial principal component analysis using the bile acid data alone revealed a partial segregation of treatment groups and controls, and this separation was further enhanced by a partial least-squares discriminant analysis (PLS-DA) as shown in Fig. 4a,b. To avoid overfitting, permutation tests with 100 iterations were performed to validate the model^30^ and the validation plots indicated the original model were valid. These data indicated that model was of modest quality and provided accurate predictions. Analysis of the animal latent variable 1 (LV1) scores for both serum (Fig. 4a) and liver (Fig. 4b) showed that bile acid levels in Geniposide treated groups differed from control at both the 100 mg/kg (*p* < *0.05*) and 300 mg/kg (*p* < *0.001*) dose. The variable importance in projection (VIP) values were used to identify the potential markers (Fig. 4c,d), and a VIP value above 1.0 was used as a cut off to select potential markers^31^. Using this criteria, we identified the bile acids TCA, TCDCA, T-α-MCA in sera and TCA, TCDCA, taurohyodeoxycholic acid (THDCA), hyodeoxycholic acid (HDCA), T-α-MCA in liver as potential markers.

### Geniposide affected sera bile acid compositions

**Figure 5:**
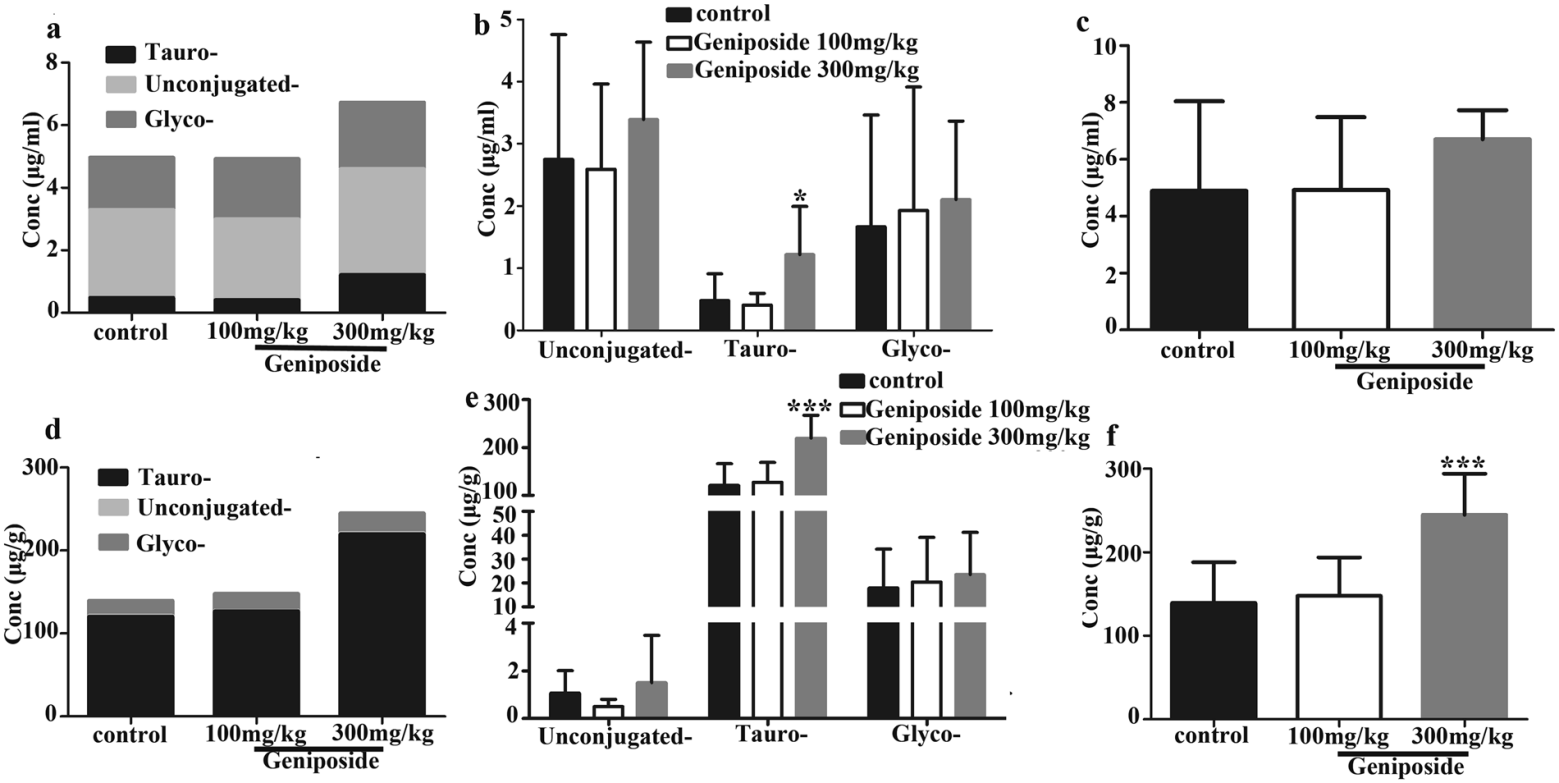
Alterations in the composition of bile acids in sera and liver after rats were treated with Geniposide. Serum proportions (**a**) and concentrations (**b**) of t-CBAs, g-CBAs and UCBAs, and concentrations of TBAs (**c**) in different groups. In addition, proportions (**d**) and concentrations (**e**) of t-CBAs, g-CBAs and UCBAs, and concentrations of TBAs (**f**) in liver of different groups. Data are presented as M ± SD of 7–8 rats. *p < *0.05*, ***p < *0.001*, compared with the control group.

TBAs, UCBAs and CBAs (including t-CBAs and g-CBAs) in serum of each rat were calculated respectively. As shown in Fig. 5a, UCBAs accounted for the largest portion of TBAs in rat sera in all groups, and no difference in serum TBAs were detected between the control and either the low or high dose of Geniposide, although an increase in TBAs was weakly indicated in the high dose versus control groups (*p* = *0.081*) (Fig. 5c). Nevertheless, t-CBAs but not g-CBAs were clearly increased (*p* < *0.05*) (Fig. 5b) after rats were treated with high, but not the low dose of Geniposide. Specifically, the high dose of Geniposide elevated the amounts of T-α-MCA, TCDCA, TCA and β-MCA (*p* < *0.05*, vs control group) by 132%, 177%, 418% and 145%, while decreasing HDCA by 71% (*p* < *0.05*) (Table 2). Notably, an increase of TCDCA and a decrease of HDCA were also observed in the 100 mg/kg Geniposide group (*p* < *0.05*) (Table 2). These results indicated that, treatment with Geniposide for 3 days could cause different changes of bile acid compositions depending on the different doses.

**Table 2:** Concentrations of bile acids in sera and liver after rats treated with Geniposide for 3 days.

	control	Geniposide	
	—	100 mg/kg	300 mg/kg
**Sera (μg/ml)**			
T-α-MCA	0.0627 ± 0.0551	0.0413 ± 0.0294	0.145 ± 0.0772*
THDCA	0.183 ± 0.203	0.138 ± 0.0539	0.126 ± 0.0355
TCA	0.138 ± 0.133	0.123 ± 0.0937	0.712 ± 0.571*
TCDCA	0.0448 ± 0.0259	0.0669 ± 0.0152*	0.124 ± 0.0260***
TDCA	0.0525 ± 0.0433	0.0387 ± 0.0295	0.109 ± 0.0987
GUDCA	0.0611 ± 0.00289	0.0612 ± 0.00328	0.0615 ± 0.00155
GHDCA	0.445 ± 0.633	0.186 ± 0.232	0.0735 ± 0.0558
GCDCA	0.0597 ± 0.0648	0.0380 ± 0.0453	0.0405 ± 0.0292
GDCA	0.119 ± 0.137	0.0861 ± 0.140	0.0750 ± 0.0595
GCA	0.988 ± 1.078	1.557 ± 1.655	1.871 ± 1.147
β-MCA	0.460 ± 0.434	0.788 ± 0.486	1.128 ± 0.330**
CA	0.555 ± 0.781	0.886 ± 0.744	1.477 ± 1.256
UDCA	0.0817 ± 0.0547	0.0447 ± 0.0249	0.0576 ± 0.0255
HDCA	1.492 ± 1.066	0.619 ± 0.182*	0.427 ± 0.123*
CDCA	0.0903 ± 0.147	0.105 ± 0.0939	0.135 ± 0.142
DCA	0.137 ± 0.0630	0.146 ± 0.109	0.165 ± 0.103
**Liver (μg/g)**			
T-α-MCA	36.112 ± 15.073	36.983 ± 16.058	61.974 ± 14.241**
THDCA	20.798 ± 10.187	11.927 ± 4.966*	7.730 ± 2.746**
TCA	47.005 ± 21.610	65.257 ± 24.042	126.849 ± 32.844***
TCDCA	4.269 ± 2.075	3.785 ± 1.578	11.199 ± 6.825*
TDCA	12.250 ± 8.509	8.903 ± 3.776	11.964 ± 8.065
GUDCA	0.173 ± 0.189	0.136 ± 0.152	0.139 ± 0.133
GHDCA	2.955 ± 3.941	1.190 ± 1.466	0.395 ± 0.523
GCA	12.660 ± 11.362	17.873 ± 16.076	21.914 ± 16.195
GCDCA	0.393 ± 0.499	0.291 ± 0.402	0.349 ± 0.301
GDCA	1.754 ± 1.608	0.949 ± 0.995	0.755 ± 0.732
β-MCA	0.413 ± 0.495	0.288 ± 0.235	0.595 ± 0.376
CA	0.145 ± 0.167	0.0876 ± 0.0864	0.779 ± 1.624
HDCA	0.470 ± 0.420	0.0817 ± 0.0324*	0.0804 ± 0.0346*
CDCA	0.0437 ± 0.0270	0.0363 ± 0.0120	0.0429 ± 0.0259

Data are presented as means ± SD concentrations in sera and liver measured using UPLC-MS/MS of 7–8 rats. *p < *0.05*, **p < *0.01*, ***p < *0.001*, compared with the control group of same bile acid.

### Geniposide affected liver bile acid compositions

Hepatic TBAs, CBAs and UCBAs results are shown in Fig. 5d–f. The t-CBAs accounted for the greatest portion of hepatic TBAs in Geniposide treated and control groups, with UCBAs and g-CBAs representing minor components (Fig. 5d). Treatment with low dose of Geniposide did not affect hepatic TBAs. As in sera, high dose of Geniposide elevated liver TBAs and t-CBAs, but not g-CBAs and UCBAs (Fig. 5e,f). The level of hepatic TBAs and the sum of t-CBAs in Geniposide 300 mg/kg group was 75% and 82% higher than controls, respectively. Treatment with high dose of Geniposide also increased multiple t-CBAs, including T-α-MCA, TCA, TCDCA (36.1, 47.0, 4.27 μg/g in control group vs 62.0, 127, 11.2 μg/g in Geniposide high dose group, respectively). THDCA and HDCA, however, were decreased by high dose Geniposide treatment. Decreases of THDCA and HDCA were also noted in low dose group (Table 2). Together these results indicated that high dose of Geniposide can cause the accumulation of bile acids in liver, mostly t-CBAs, that could be related to liver injury.

### Geniposide impact on hepatic bile acid transport and metabolism and gene expression

**Figure 6:**
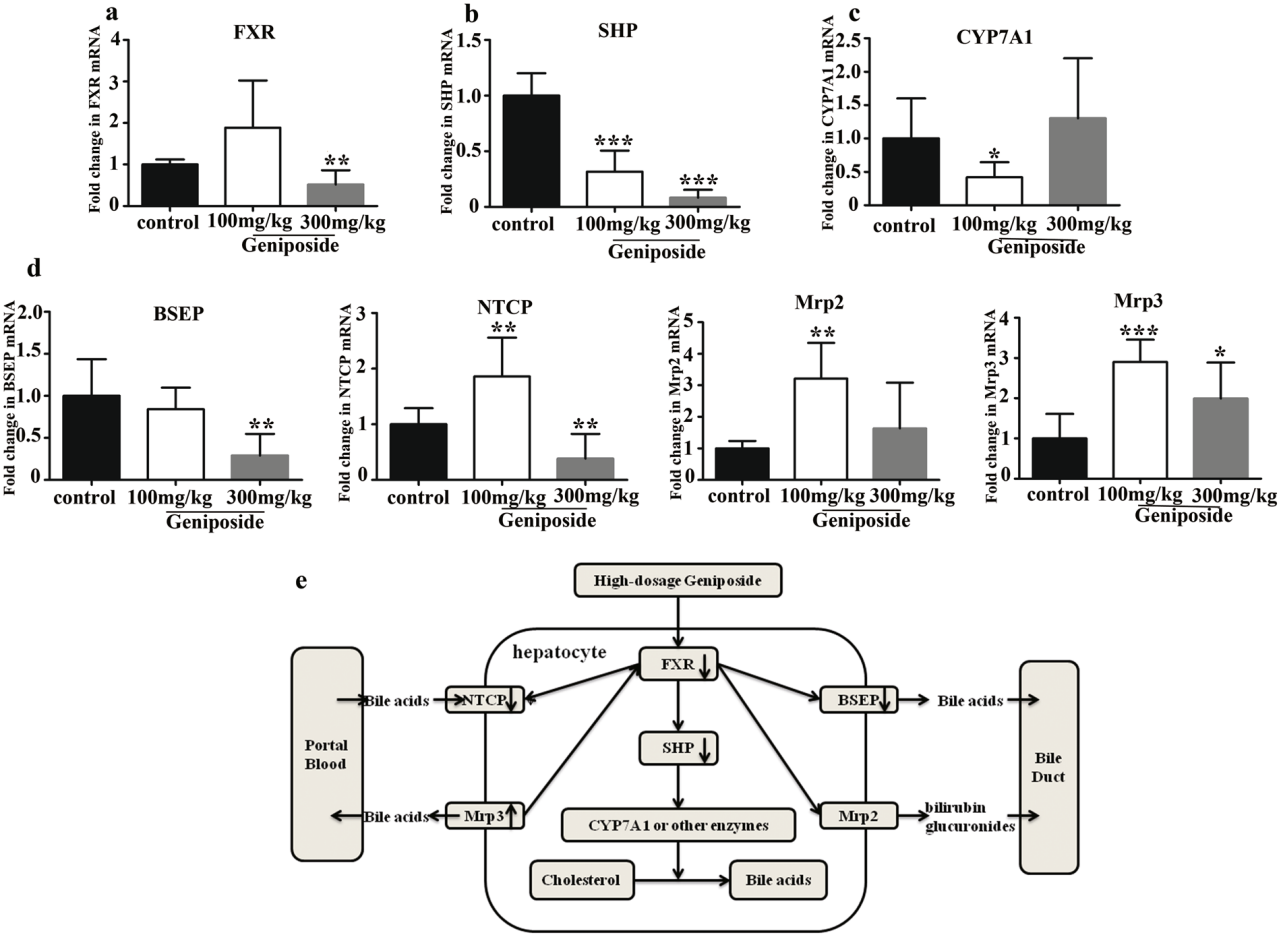
Expressions of genes involved in hepatic bile acids regulation. Quantitative real-time PCR analysis was performed to measure the expressions of genes in livers, including FXR (**a**), SHP (**b**), CYP7A1 (**c**) and genes of multiple transporters involved in bile acids transportation (**d**): BSEP, NTCP, Mrp2 and Mrp3. Diagrammatic sketch of the modulation of hepatic gene expressions involved in bile acid synthesis and transportation associated with high dose of Geniposide-induced liver injury (**e**). The “up arrow” and the “down arrow” represented up-regulation and down-regulation of high-dosage Geniposide (300 mg/kg) respectively. Data are presented as M ± SD of 7–8 rats. *p < *0.05*, **p < *0.01*, ***p < *0.001*, compared with the control group.

To understand the mechanism of the Geniposide on bile acid metabolism associated with hepatotoxicity, we used quantitative real-time PCR to analyze the gene expressions of a nuclear bile acid receptor (FXR), an enzyme for bile acid synthesis cholesterol 7α-hydroxylase (CYP7A1) and atypical nuclear receptor SHP. As shown in Fig. 6a, the expression of FXR mRNA was suppressed by high dose Geniposide (*p* < *0.01*), but potentially upregulated by low dose (*p* = *0.1*). Figure 6b showed that both high dose and low dose of Geniposide suppressed SHP mRNA expression (*p* < *0.001*). The expression of CYP7A1 mRNA suppressed at the 100 mg/kg dose (*p* < *0.05*) but unaffected at the 300 mg/kg dose of Geniposide (*p* = *0.3*) (Fig. 6c). Multiple changes in genes involved in bile acid transport were also observed. As shown in Fig. 6d, high dose of Geniposide inhibited the expression of BSEP mRNA (*p* < *0.01*). The expression of NTCP mRNA was down-regulated by high dose of Geniposide, but up-regulated by low dose of Geniposide (*p* < *0.01*), following a pattern similar to the FXR response. Only the low dose showed a clear up-regulation on Mrp2 mRNA expression (*p* < *0.01*), while the expression of Mrp3 mRNA was clearly up-regulated by both high and low doses of Geniposide (*p* < *0.05*). The results indicated that different doses of Geniposide result in distinct effects on the genes involve in bile acid synthesis, transportation and secretion, and compensatory mechanisms induced by the low dose may be overwhelmed by Geniposide-induced liver injury at the high dose.

## Discussion and Conclusion

The occurrence of hepatotoxicity cases linked to CMM have raised serious concerns regarding CMM safety^32^. CMM taken at recommended doses by the Chinese Pharmacopoeia generally do not cause liver injury, but increasing the dosage of some CMM may lead to hepatotoxicity^33^. Moreover, the concentration of active ingredients in an herb can be diverse due to differences in growth areas, harvest time, and processing method and so on. Hence, even if people consume the same amount of an herb, the intake of active ingredients could differ.

A major active constituent in FG, Geniposide, is a critical marker for FG quality^3^. In the present study, we found that Geniposide could cause distinct liver injury in rats at a dose of 300 mg/kg, without measurable hepatotoxicity at 100 mg/kg. Other studies have also revealed hepatotoxicity at high-dosage of Geniposide (≥280 mg/kg)^7, 33^, supporting dose-dependent Geniposide-induced hepatotoxicity. Geniposide has been reported to have various pharmacological effects, being especially protective against hepatic injury caused by alcohol, high fat diet or carbon tetrachloride at the dose range 25–100 mg/kg^10, 34^ in rats. While Geniposide causes hepatotoxicity at doses several times higher than the doses used to elicit these pharmacological effects in the experiments, the potential for patients to be exposed to high doses of Geniposide in the clinic should be a concern since the minimum content of Geniposide in FG is established at 1.8% but no upper level is defined by the China Pharmacopeia^3^. Nevertheless, the content of Geniposide in FG is influenced by several factors, such as growing areas, processing procedure, and even collection time^11–13^ and the highest content is ~6% which is 3–4 times the minimum standard. So, even though the same doses of FG is taken by patients this could represent substantial differences in Geniposide exposure. The highest daily dose of FG for human is 10 g recommended in China Pharmacopeia^3^, that may be equivalent to 180 mg to 600 mg (3 mg/kg to 10 mg/kg for 60 kg human) of Geniposide corresponding to the range of content of Geniposide in FG (1.8% to 6%). According to the dose conversion method between animal and human^35^, doses of Geniposide 100, 300 mg/kg used in rats in this study could be converted to estimate human equivalent dose (HED) 16, 48 mg/kg respectively, and the HED is divided by a factor value of 10 to obtain the pharmacologically active doses for humans (1.6, 4.8 mg/kg). It is known that the pharmacologically active doses for humans (4.8 mg/kg) is within the daily dose range of Geniposide in FG in human mentioned above. Thus, hepatotoxicity due to Geniposide at 300 mg/kg may be relevant for humans. There is a possibility for patients with a risk of hepatotoxicity when FG has a high content of Geniposide. Based on this study, we suggest that the quality control standard for the content of Geniposide in the herb of FG should have both upper and lower limitation values to prevent hepatotoxic events.

The mechanism of Geniposide-induced hepatotoxicity has not been elucidated, though oxidative stress was postulated^7, 8^. After treatment with 300 mg/kg Geniposide, the serum ALP and γ-GT were obviously increased, both of which have been used as markers of the cholestasis^36^. The increase of serum ALP and γ-GT could be a side effect of many medications^17^ as they are general reporters of liver damage. Therefore, we performed further tests on the bile acids in sera and livers, and found that there were significant changes in the compositions of serum and liver bile acids following treatment with Geniposide 300 mg/kg. Our results revealed that disturbances in bile acid formation or secretion may be involved in Geniposide-induced hepatotoxicity.

Bile acids are endogenous molecules that normally regulate cholesterol homeostasis, lipid solubilization and metabolism^37^. Abnormally high concentrations of bile acids, such as occurring cholestasis, can result in intrahepatic accumulation of toxic bile acids leading to hepatic damage by producing pathophysiological effects including mitochondrial dysfunction with overgeneration of reactive oxygen and nitrogen species^19, 38, 39^. Moreover, even minor liver damage can cause the perturbation of serum and hepatic bile acids^40^. Various liver disorders such as nonalcoholic fatty liver disease (NAFLD), drug-induced liver injury could increase the levels of bile acids in liver^41^. Therefore, bile acids are considered as highly sensitive markers for liver injury and liver dysfunction, and used as potential biomarkers in drug-induced liver injury^42^.

In this study, we investigated the bile acid profiles in both sera and livers of the rats with or without Geniposide treatment. Multivariate discriminant analyses^43^ showed clear differences between high-dosage Geniposide (300 mg/kg) and control group, but weak difference between the control group and the low-dosage Geniposide (100 mg/kg) group. Our study revealed that Geniposide-induced hepatic injury was associated with the change of bile acids in sera and livers. Concurrent with liver injury, TBAs, especially the dominant types of bile acids^41^ and the t-CBAs, markedly increased in the livers after rats were treated with high-dose of Geniposide. Among t-CBAs, TCDCA and taurodeoxycholic acid (TDCA) have postulated as inducers of cholestasis that significantly elevate serum levels of ALT and AST in rats^44^. Additionally, TCA, TCDCA and TDCA are substantially elevated in acetaminophen-induced acute liver failure patients^45^. Strong correlations were noted between hepatic necrosis and the bile acids TCA and TDCA in an acetaminophen‑induced rat liver injury model^46^. Here, TCA, TCDCA and T-α-MCA were increased in both sera and livers, and were the strongest bile acid discriminators of dose, suggesting them as valuable serum potential markers for Geniposide-induced liver injury in rats.

Correlation coefficients (r) between variables of bile acids and ALP, γ-GT in serum, which are commonly used biomarkers in evaluating drug-induced choletasis^36^, were calculated^47^(Supplementary Table S1). The correlation analysis suggested significant positive correlations between concentrations of major t-CBAs (T-α-MCA, TCDCA, TCA, TDCA), partial UCBAs (β-MCA, CDCA, CA) and GCA in serum and ALP, γ-GT. In addition, ALP, γ-GT positively correlated significantly with the concentrations of major t-CBAs (T-α-MCA, TCDCA, TCA) and CA in liver. Therefore, the results revealed the concentrations alteration of t-CBAs in particular could have a relationship with high dose Geniposide-induced liver injury.

Bile acid homeostasis is tightly regulated via a feedback loop operated by FXR and SHP^48^. The hepatic FXR induces SHP in liver leading to inhibition of CYP7A1, the rate-limiting enzyme in bile acid synthesis^24, 37^. The loss of FXR and SHP can rapidly result in cholestasis and liver injury^48^. As we observed in this study, high-dose of Geniposide (300 mg/kg) significantly down-regulate the expression of FXR and SHP mRNA, and SHP downregulation was observed at the lower dose as well (Fig. 6e). However, increased bile acid production was only weakly suggested (*p* = *0.3*) by increased CYP7A1 expression with 300 mg/kg Geniposide exposure, suggesting other mechanisms must be at work to elevate hepatic bile acid concentrations to promote liver injury.

Disruption in bile acid export could also lead to their elevations in the liver and we found that multiple hepatocytes transporters were involved in Geniposide-induced bile acid increase and liver injury (Fig. 6e). BSEP is the major transporter for the secretion of bile acids from hepatocytes into bile^49^, and BSEP inhibition is a known risk factor for drug-induced cholestatic hepatotoxicity thought to play an important role in the development of liver injury^50^. Geniposide at 300 mg/kg down-regulated BSEP mRNA expression in the liver which would support the accumulation of bile acids in hepatocytes. The transport of bile acids across the basolateral membrane of the hepatocytes is mainly mediated by the NTCP. Geniposide at 300 mg/kg also suppressed hepatic NTCP mRNA expression which could be a negative feedback mechanism to reduce bile acid entry in response to elevated hepatocyte bile acid concentrations^49^. Mrp2, located in the canalicular membrane of hepatocytes, transport bile acids from the hepatocytes into the bile^49^. Mrp3 is localized to the basolateral membrane of the hepatocytes mediating the export of bile acids. Geniposide was shown to up-regulate the expression of Mrp2 mRNA and Mrp3 mRNA in rat livers, significantly at doses 100 mg/kg (on Mrp2) and 100, 300 mg/kg (on Mrp3). The up-regulation of Mrp3 could be a compensatory action for bile acid efflux when BSEP-mediated biliary excretion is impaired^51^, to reduce the accumulation of bile acids in hepatocytes. The elevation of Mrp2 could facilitate hepatic bile acids into the canaliculus, and thus reduce the risk of liver injury. In addition, Mrp2 mediates the export of bilirubin conjugates from hepatocytes^52^, consistent with the Geniposide-induced bilirubin decrease in this study. In comparison, Geniposide had more vigorous effect in up-regulation of Mrp3 genes at dose of 100 mg/kg rather than that at dose of 300 mg/kg. One possibility for this observation would be an accumulating hepatocyte damage that is reducing the livers ability to sustain an effective compensatory defense via Mrp2 and Mrp3 induction, and is consistent with the higher levels of bile acids and liver injury observed at high Geniposide dose.

In conclusion, high dose Geniposide can cause liver injury which is associated with, and potentially linked to increase of bile acid concentrations in hepatocytes. These changes appeared weakly associated with an increase of bile acid synthesis due to CYP7A1 dysregulation, with strong suppression of FXR and SHP. Clear dose dependent impacts on hepatic bile acid excreting gene expression were identified. While reductions in bile acid excretion through the primary route regulated by BSEP associated with low-dose Geniposide appeared to be effectively compensated for by shifts in NTCP, Mrp2 and Mrp3 expression, these systems could not prevent hepatic bile acid accumulation as well as liver injury caused by high dose of Geniposide. Based on the results, we assume that high dose of Geniposide-induced rat liver injury was likely cholestatic, and TCA, TCDCA and T-α-MCA are potential serum potential markers for Geniposide-induced liver injury in rats.

## Methods

### Ethics statement

This study was approved by the Research Ethics Committee of the Institute of Chinese Materia Medica, China Academy of Chinese Medical Sciences, (ICMM, CACMS), Beijing China. The experiment was carried out in accordance with the ethical guidelines and regulations for the use of laboratory animals. All animal-related procedures adhered to the protocol, which was approved by the Institutional Animal Care and Use Committee of the ICMM, CACMS.

### Drug and chemicals

Geniposide, the purity 97.12% (HPLC), was purchased from Beijing Saibaicao technology Co., LTD (Beijing, China). All reference bile acids purchased were of high purity. TCA and CUDA were purchased from Cayman chemical company (Ann Arbor, Michigan, US). T-α-MCA, TDCA, glycoursodeoxycholic acid (GUDCA), glycohyodeoxycholic acid (GHDCA) and β-MCA were purchased from Toronto Research Chemicals (Toronto, Canada). glycochenodeoxycholic acid (GCDCA), glycodeoxycholic acid (GDCA) and glycocholic acid (GCA) were purchased from Nanjing Shenglide Technology Co., LTD (Nanjing, China). TCDCA, THDCA, CA, UDCA, HDCA, CDCA and DCA were purchased from national institutes for food and drug control (NIFDC, Beijing, China).

### Animals and experimental procedure

Specific pathogen free male Sprague-Dawley rats (24) provided by Vital River Laboratory Animal Technology Co. Ltd. (Beijing, China) were received at 10 wks of age, with body weights ranging from 200–220 g. Animals were housed in an environmentally-controlled animal facility with room temperature 23 ± 3 °C, relative humidity of 40~70%, air ventilation of approximate 15 times/hr and a 12-hour light/dark cycle. The animals were allowed filtered tap water and the fixed-formula rat granular feed ad libitum.

The rats were randomly divided into Geniposide 0, 100, 300 mg/kg^35, 53^ groups. Rats were dosed by a gastric gavage once daily for three consecutive days while the rats in control group received an equal volume of pure water. Twenty-four hours after the last administration, all rats were anesthetized with sodium phenobarbital by intraperitoneal injection under the condition of fasting overnight and the blood samples were collected from the abdominal aorta and euthanized by exsanguinations, and then livers were dissected. The sera were prepared by centrifugation at 3,000 rpm for 15 min after coagulating at room temperature for analysis of biochemical parameters and bile acids. A portion of liver was preserved in neutral buffered formalin for histopathological examination, while the remaining portion was stored at −80 °C for further analysis of bile acids by LC-MS/MS and gene expression by quantitative real-time PCR.

### Biochemical assay

Serum biochemistry analysis, including AST, ALT, ALP, γ-GT, CHO, TBIL was assayed by using TBA-40FR antomatic biochemistry analyzer (Toshiba, Japan).

### Histopathological examination

Liver samples were routinely fixed with neutral buffered formalin, and embedded in paraffin. Four micron thick sections were cut and stained with HE. The histomorphology was examined under the light microscopy (Olympus, Japan).

### Analysis of bile acids in serum and liver by UPLC-MS

A 100 μL aliquot of serum sample was added to washed and activated SPE columns (Waters Oasis HLB 1cc, 10 mg). While in the SPE reservoir, the serum was spiked with 5 μL anti-oxidant solution (0.1 mg/ml solution BHT/EDTA in 1:1 MeOH: water) and diluted to 1 column volume with 5% MeOH w/0.1% acetic acid (v/v. Samples were loaded by gravity and washed with 1 column volume of 30% MeOH w/0.1% acetic acid (v/v). Sample extracts containing bile acids were eluted into 2 mL vials containing 10 μL of 20% glycerol solution in MeOH using 0.2 mL MeOH, followed by 0.5 mL acetonitrile (ACN), followed by 0.7 mL ethyl acetate. Solvents were removed under nitrogen and the residual 2 μL glycerol was redissolved with 100 μL of 100 nM 1-cyclohexylureido, 3-dodecanoic acid (CUDA; Cayman Chemical, Ann Arbor MI, USA) internal standard (in 50:50 MeOH: ACN) to tubes. Samples were filtered at 0.1 µm by centrifugation through Durapore PVDF membranes (Millipore) for 3 min at 6 °C at 4500 g (rcf) and stored at −20 °C for less than 1 wk prior to LC-MS/MS.

The pulverized liver (15 mg) was placed into a tared and cleaned polyproylene tube, spiked with 5 μL anti-oxidant solution, and mixed with 500 μL MeOH, followed by 30 sec vortex-mixing. After centrifugation at 10,000 g for 5 min at room temperature, the supernatant was collected, spiked with glycerol, dried and then reconstituted in 100 μL CUDA, filterd and stored as described above. The quality control samples were kept at −80 °C and the calibration samples were kept 4 °C until analyzed.

A Waters Acquity UPLC System coupled with API 5500 QTRAP mass spectrometry (AB Sciex) was used for the quantification of Bas. The UPLC system consists of a binary pump, a continuous vacuum degasser, a thermostated auto-sampler and a column compartment. Chromatographic separation of bile acids was carried out on an ACQUITY UPLC BEH column (2.1 × 100mm, 1.7μm) (Waters Corp., Milford, US). The mobile phase made up of 0.1% formic acid in water (A) and 0.1% formic acid and acetonitrile (B). The gradient elution was as follows: 90%A (0–0.5 min), 90–75%A (0.5–1.0 min), 75–60%A (1.0–11.0 min), 60–5%A(11.0–12.5 min), 5%A (12.5–14.0 min), 5–90%A (14.0–14.5 min), and 90%A (14.5–16.0 min). The flow rate of mobile phase was 0.4 mL/min and the injection volume was 5 μL. The mass spectrometer was operated in the ESI negative mode with multiple reaction monitoring (MRM) function for the quantitation^54, 55^ and more details on the MRM conditions were shown in Supplementary Table S2. The temperature of ion source was set up at 600 °C. The total chromatographic operation was divided into several periods. The ion dwell time and transition about all of the compounds were set reasonably. Data were manipulated with SIMCA-P software, Version 12.0.

Instrument responses were calibrated with a mixture of 16 bile acids in methanol. The linear regression parameters obtained for each bile acid were showed in Supplementary Table S3. The accuracy was evaluated by the analysis of carbon-stripped serum spikes at low and high concentrations (Supplementary Table S4).

### Quantitative Real-time PCR analysis

Total hepatic RNA was extracted by using a total RNA kit (OMEGA, Georgia, U.S.A) according to manufacturer instructions. An aliquot of 1 μg RNA was applied for reverse transcription with oligo-dT primer (TOYOBO, OSAKA, Japan). Quantitative real-time PCR was performed using the Roche 480 instrument (Roche, Mannheim, Germany) and SYBR Green PCR Master Mix (Roche, Mannheim, Germany) for the subsequent genes with the corresponding primers (Sangon Biotech, Beijing, China) (Supplementary Table S5). Quantification was performed by the ΔΔCT method. The quantity of mRNA was normalized with the internal standard GAPDH.

### Statistical analyses

The data are expressed as the mean (M) ± standard deviation (SD). All data are independent samples. Statistical analysis of measurement data was performed using Student’s *t* test and person correlation coefficient (r) was performed using correlation analysis with SPSS statistical software, version 16.0. Bile acid data were also used to perform the PLS-DA using SIMCA-P v.12.0 (Umetrics, San Jose, US). The data of Geniposide treatment groups were compared with control group, and a p-value of <0.05 was considered to be statistically significant.

## Electronic supplementary material

supplementary information
