# Supplementary material for: Dose-related liver injury of Geniposide associated with the alteration in bile acid synthesis and transportation
Source: Sci Rep. 2017 Aug 21;7:8938. doi: 10.1038/s41598-017-09131-2 (PMC5566417; doi:10.1038/s41598-017-09131-2)
Supplement: Supplementary file 1 — supplementary information [file 41598_2017_9131_MOESM1_ESM.pdf]

Dose-related liver injury of Geniposide associated with the alteration in bile acid synthesis and transportation

JingzhuoTian[1#], Jingjing Zhu[1#], Yan Yi[1#], Chunying Li[1], Yushi Zhang[1], Yong Zhao[1], Chen Pan[1], Shixie Xiang[1], Xiaolong Li[1],Guiqin Li[1], John W Newman[2,3,4], Xiaoyi Feng[1], Jing Liu[1], Jiayin Han[1], Lianmei Wang[1], Yue Gao[5], Michael R. La Frano[2,6], Aihua Liang[1*]

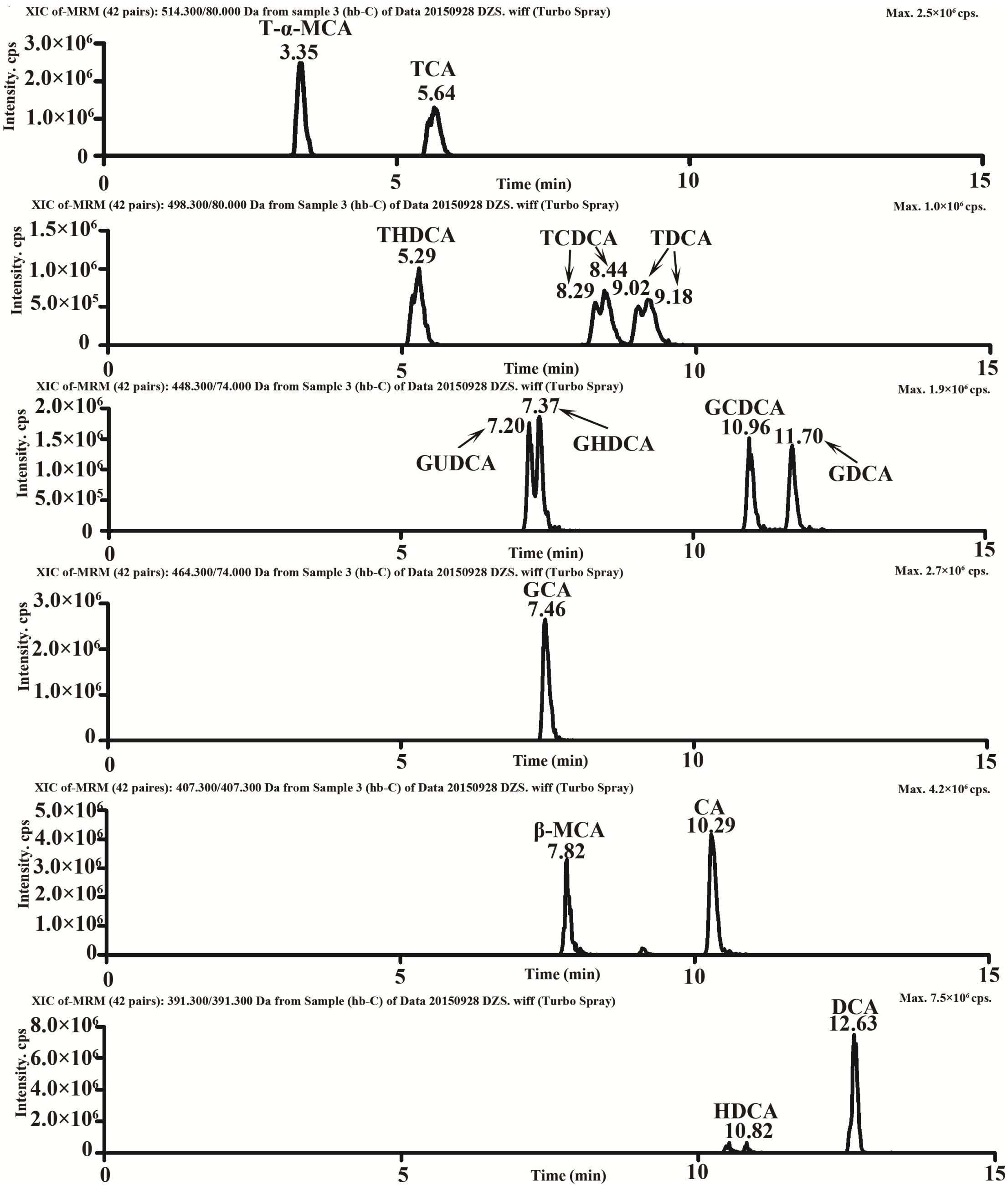

Supplementary Figure S1

Representative UPLC-MS/MS chromatogram authentic standard of bile acids.

Supplementary Table S1

Results of the correlation analysis consist of correlation coefficient.

Bile acid	ALP (serum)		γ-GT (serum)		ALP (liver)		γ-GT (liver)	
	r	*p*	*r*	*p*	*r*	*p*	*r*	*p*
T-α-MCA	0.638	0.000***	0.533	0.002**	0.523	0.003**	0.366	0.043*
TCDCA	0.667	0.000***	0.569	0.001**	0.838	0.000***	0.676	0.000***
TCA	0.633	0.000***	0.534	0.002**	0.659	0.000***	0.436	0.014*
THDCA	0.296	0.106	0.264	0.151	-0.183	0.324	-0.208	0.261
TDCA	0.492	0.005**	0.379	0.035*	-0.036	0.848	-0.203	0.274
GCA	0.394	0.028*	0.368	0.042*	0.187	0.315	0.112	0.549
HDCA	-0.123	0.510	-0.164	0.379	-0.047	0.800	-0.272	0.139
β-MCA	0.698	0.000***	0.475	0.007**	0.312	0.113	-0.083	0.680
CA	0.693	0.000***	0.535	0.002**	0.714	0.000***	0.451	0.011*
CDCA	0.396	0.027*	0.364	0.044*	0.439	0.014*	0.290	0.113
DCA	0.163	0.380	0.113	0.544	—	—	—	—

r: person correlation coefficient

*$P<0.05$, **$P<0.01$, ***$P<0.001$, compared with the control group.

Supplementary Table S2

The MRM acquisition parameters of bile acids and internal standards

Compounds	Parent ion (m/z)	Daughter ion (m/z)	DP (V)	CE (V)
β-MCA	407.3	407.3	115	30
CA	407.3	407.3	125	30
UDCA	391.3	391.3	125	30
HDCA	391.3	391.3	125	30
CDCA	391.3	391.3	130	30
DCA	391.3	391.3	130	30
LCA	375.3	375.3	130	35
T-α-MCA	514.3	80.0	155	110
THDCA	498.3	80.0	145	110
TCA	514.3	80.0	185	115
TCDCA	498.3	80.0	145	110
TDCA	498.3	80.0	140	110
GUDCA	448.3	74.0	115	70
GHDCA	448.3	74.0	120	70
GCA	464.3	74.0	125	70
GCDCA	448.3	74.0	125	65
GDCA	448.3	74.0	125	65
CUDA	341.3	216.2	65	35

The MRM acquisition parameters of bile acids and internal standards including Q1, Q3, declustering potential (DP) and collision energies (CE) were showed.

Supplementary Table S3

Linearity Data and Quantitation Ranges Obtained for Bile acids

Bile acids	linearity	Correlation coefficient （R）	Range （nM/L）	LOD（nM/L）
β-MCA	y = 0.242x + 2.5985	0.9999	1.1102~3700.7122	0.7091
CA	y = 0.5622x + 24.854	0.9993	0.7807~8674.4010	0.7203
UDCA	y = 0.1811x + 1.1484	0.9977	0.4979~1659.8314	0.3910
HDCA	y = 0.108x + 6.8202	0.9991	0.8021~8912.0412	0.5539
CDCA	y = 0.1711x + 3.1875	1	0.3460~3843.9005	0.1990
DCA	y = 0.5679x + 18.012	0.9989	0.3439~3820.9746	0.2793
LCA	y = 0.0206x + 2.2645	0.9984	0.8398~9331.5984	0.8398
T-α-MCA	y = 0.1368x + 3.1354	0.9995	1.4917~4972.3404	1.4689
THDCA	y = 0.2012x + 1.0175	0.9999	0.6062~2020.7403	0.5885
TCA	y = 0.1428x - 0.7775	0.999	1.7613~2935.4276	1.2325
TCDCA	y = 0.2114x + 0.9555	0.9997	0.6082~2027.3503	0.5768
TDCA	y = 0.6199x - 0.5963	0.999	0.3452~575.2842	0.3024
GUDCA	y = 0.2132x - 0.9757	0.9971	0.7009~1168.1197	0.5156
GHDCA	y = 0.268x - 0.0535	0.9999	0.7041~1173.4909	0.3388
GCA	y = 0.17x + 0.2256	0.9999	1.9631~3271.7667	0.8608
GCDCA	y = 0.2152x - 1.1602	0.999	0.6719~1119.8872	0.5714
GDCA	y = 0.183x - 0.8729	0.9978	0.4027~671.2335	0.3678

Supplementary Table S4

Concentrations and recoveries of bile acids in sera after rats treated with Geniposide for 3 days.

Bile acids	Concentration (ng/ml)	Recovery (%)	Bile acid	Concentration (ng/ml)	Recovery (%)
β-MCA	9800	76.7±0.1	TCA	9997	55.1±0.1
	98	97.0±0.1		99.97	60.6±0.4
CA	10040	85.6±0.3	TCDCA	5004	54.4±0.1
	100.4	79.8±0.1		50.04	69.4±0.4
UDCA	10008	71.3±0.1	TDCA	10020	66.8±0.1
	100.8	77.1±0.2		100.0	87.5±0.2
HDCA	5010	91.4±0.2	GUDCA	9980	79.1±0.2
	50.1	81.8±0.2		99.8	48.1±0.4
CDCA	5010	86.8±0.3	GHDCA	9960	104.0±0.1
	50.1	103.4±0.1		99.6	90.4±0.2
DCA	5000	92.6±0.1	GCA	10020	98.4±0.1
	50	106.1±0.1		100.02	98.9±0.1
T-α-MCA	9996	74.3±0.1	GCDCA	5020	99.6±0.2
	99.6	76.2±0.4		50.2	88.8±0.1
THDCA	9996	72.4±0.1	GDCA	9989	102.7±0.1
	98.96	75.6±0.1		99.89	88.6±0.1

Method performance was evaluated by analysis of carbon-stripped serum spikes at low and high concentrations.

Supplementary Table S5

PCR for the subsequent genes with the corresponding primers.

Gene	Primer
FXR	Forward: 5’-CAGCAGACCCTCCTGGATTA-3’<br>Reverse: 5’-TCTTCGTGGTCCAGTGTCTG-3’
SHP	Forward: 5’- CCTTGGATGTCCTAGGCAAG-3’<br>Reverse: 5’-CACCACTGTTGGGTTCCTCT-3’
CYP7A1	Forward: 5’-TGCCTTCTGTTACCGAGTGATGTT-3’<br>Reverse: 5’-ACCGGCAGGTCATTCAGTTGCACT-3’
BSEP	Forward: 5’-TCTATGGACTCTGCTTTGCCTTTT-3’<br>Reverse: 5’-GCCCAGACCTTCGTAGGCTA-3’
NTCP	Forward: 5’-CCCTGATGCCCTTCTCTGG-3’<br>Reverse: 5’-GAATCCTGTTTCCATGCTGATG-3’
Mrp2	Forward: 5’-TGGAGTTGGCTCACCTCAGATC-3’<br>Reverse: 5’-CTAGAGCTCTGTGTGATTCACATTTTCA-3’
Mrp3	Forward: 5’-GTGCTGAAGAATTTGACTCTG-3’<br>Reverse: 5’-GACCAGGACCCGGTTGTAGTC-3’
GAPDH	Forward: 5’-CCTGGAGAAACCTGCCAAGTAT-3’<br>Reverse: 5’-AGCCCAGGATGCCC TTTAGT-3’
